# Predictive factors of noninvasive ventilation failure in a pediatric ICU

**DOI:** 10.1186/cc14703

**Published:** 2015-09-28

**Authors:** Marcela B Alith, Lilian AY Fernandes, Rodrigo C Borges, Regina HA Quinzani, Dumara N Oliveira, Andréia M Ferreira, Nilce C Oliveira, Alexandra S Colombo

**Affiliations:** 1 University Hospital, University of São Paulo, Brazil; 2 Federal University of São Paulo, Paulista School of Medicine (UNIFESP/EPM), Brazil

## Introduction

Noninvasive ventilation (NIV) has been developed to reduce complications associated with tracheal intubation and conventional mechanical ventilation. The aim of NIV is to gain control of acute respiratory failure, avoiding intubation; however, when intubation is required, its application should not be delayed, as this may result in a worse prognosis. This is the main reason to look for reliable failure signs of the technique.

## Objective

To identify failure prognostic signs of NIV in pediatric acute respiratory failure.

## Methods

This retrospective study was based on data collection from medical records of patients admitted to the pediatric ICU of University Hospital, University of São Paulo, from March to September 2012 and during the same period in 2013. Patients were divided into two groups: success group, in which patients who used NIV did not require intubation; and failure group, which included all patients who required intubation. The following variables were analyzed: age, sex, weight, personal history, previous use of oxygen, NIV success or failure, cause of failure, respiratory rate, heart rate, oxygen saturation, PRISM, PIM, the NIV devices and ventilatory parameters during NIV placement and during withdrawal.

## Results

The charts of 112 patients were analyzed, 55 in the success group and 57 in the failure group. Most children who failed (32.14 %) were male. The median PRISM value in the failure group was 7 (5-8) (*p *= 0.000) and the median time of NIV use in this group was 570 (182-1230) minutes (*p *= 0.000). In the univariate analysis, PEEP (*p *= 0.003), fraction of inspired oxygen (*p *= 0.000), oxygen saturation (*p *= 0.014), respiratory rate (*p *= 0.000), heart rate (*p *= 0.000) and need for sedation (*p *= 0.000) had a statistically significant difference in the moment of NIV withdrawal in the failure group. Logistic regression analysis showed that the independent factors significantly related to NIV failure were PRISM, total time in minutes and respiratory rate at the moment of withdrawal, considering the statistically significant value of *p *<0.05.

## Conclusion

The PRISM value, NIV duration and respiratory rate can predict NIV failure in the pediatric population.

